# Polymorphisms in the *CTLA4* promoter sequence are associated with canine hypoadrenocorticism

**DOI:** 10.1186/s40575-020-0081-4

**Published:** 2020-03-04

**Authors:** Alisdair M. Boag, Andrea Short, Lorna J. Kennedy, Hattie Syme, Peter A. Graham, Brian Catchpole

**Affiliations:** 1 Pathobiology and Population Sciences, The Royal Veterinary College, University of London, London, UK; 2 The Queen’s Medical Research Institute, Centre for Cardiovascular Science, University of Edinburgh, Edinburgh, UK; 3 Centre for Integrated Genomic Medical Research, University of Manchester, Manchester, UK; 4 Clinical Science and Services, The Royal Veterinary College, University of London, London, UK; 5 Faculty of Medicine & Health Sciences, School of Veterinary Medicine and Science, University of Nottingham, Nottingham, UK

**Keywords:** Addison’s, Canine, CTLA-4, Hypoadrenocorticism, Promoter

## Abstract

**Background:**

Canine hypoadrenocorticism is an immune-mediated endocrinopathy that shares both clinical and pathophysiological similarities with Addison’s disease in humans. Several dog breeds are overrepresented in the disease population, suggesting that a genetic component is involved, although this is likely to be polygenic. Previous research has implicated *CTLA4* as a potential susceptibility gene. CTLA4 is an important regulator of T cell function and polymorphisms/mutations in *CTLA4* have been associated with a number of autoimmune phenotypes in both humans and rodent models of autoimmunity. The aim of the current study was to undertake a case:control association study of *CTLA4* promotor polymorphisms in three dog breeds, cocker spaniels, springer spaniels and West Highland white terriers (WHWT).

**Results:**

Polymorphisms in the CTLA4 promoter were determined by PCR and sequence-based typing. There were significant associations with three promoter haplotypes in cocker spaniels (*p* = 0.003). A series of SNPs were also associated with hypoadrenocorticism in cocker spaniels and springer spaniels, including polymorphisms in predicted NFAT and SP1 transcription factor binding sites.

**Conclusions:**

This study provides further evidence that *CTLA4* promotor polymorphisms are associated with this complex genetic disease and supports an immune mediated aetiopathogenesis of canine hypoadrenocorticism.

## Plain English summary

Around one in 500 dogs are affected by Addison’s disease (hypoadrenocorticism), a lifelong, life threatening, disease that also less commonly affects humans. Addison’s disease is caused by a lack of cortisol (stress hormone) and other hormones due to destruction of the adrenal gland that makes these hormones. The most common cause of adrenal gland destruction is autoimmunity, meaning the body’s immune system attacks its own gland, similar to type 1 diabetes or rheumatoid arthritis. The reasons why this happens are not fully understood.

In this study we looked at genetic differences in dogs specifically in the area controlling expression (promotor region) of a gene, CTLA4. CTLA4 is vital to immune system function. Specifically, CTLA4 acts as a brake on the immune system, so less CTLA4 means less of a brake and therefore possibly more autoimmunity. We showed changes in this promotor region are associated with increased risk of Addison’s in springer spaniels and cocker spaniels. This highlights CTLA4 as likely one of the genetic reasons why some dogs get this disease and others do not.

By better understanding the underlying genetic reasons for Addison’s disease in dogs we aim to be able to better help dogs with the disease and hopefully help understand the human disease better too.

## Background

Canine hypoadrenocorticism, in common with human autoimmune Addison’s disease (hAAD), results from a deficiency in production of steroid hormones from the adrenal gland [1]. Clinical signs associated with adrenal insufficiency are varied and dogs may present in an ‘Addisonian crisis’ with electrolyte disturbances, collapse and shock. Hypoadrenocorticism is a potentially fatal disease, which appears to be under diagnosed [2, 3]. The pathology in the adrenal gland is consistent with an autoimmune aetiology, characterised, at least in the early stages, by immune cell infiltration, similar to that seen in hAAD [2, 4]. Furthermore, circulating autoantibodies, which can be long lived, have been demonstrated in a subset of dogs, and are linked to a susceptibility genotype [5, 6].

In man, the primary susceptibility locus associated with hAAD, is the human leukocyte antigen (HLA), encoding major histocompatibility complex (MHC) molecules [7]. Variation in other immune response genes have also been shown to be involved in susceptibility to hAAD, including the protein tyrosine phosphatase, non-receptor 22 (*PTPN22*) gene, which is involved in intracellular T cell receptor signalling [8, 9] and cytotoxic T-lymphocyte-associated protein 4 (*CTLA4*) [8, 10]. Due to its important role in immune regulation and tolerance, *CTLA4* been posited as a general autoimmune susceptibility locus by several authors [10–13]. *CTLA4* polymorphisms have been extensively studied in the human population and consistent associations have been found with hAAD [14–16] including a Europe-wide meta-analysis which demonstrated a significant association between hAAD and a SNP in exon 1, within the signal peptide of the coding sequence [17] and associations with a promoter polymorphism and hAAD in the Norwegian population [16, 18]. However, the relationship between CTLA4 polymorphisms and hAAD is not found in all ethnic groups [14, 19] and it is likely that within different populations, different polymorphisms exert different effects within a complex genetic landscape [18, 20].

In dogs, genetic susceptibility to hypoadrenocorticism has been evidenced through breed predispositions and further revealed by pedigree analyses [21–26]. More focussed dissection of underlying genetic factors has shown dog leukocyte antigen (DLA) class II variation to be significantly associated with hypoadrenocorticism [5, 27–29]. Whilst the significance of these findings have been contested [30, 31] it is the case that fine mapping techniques have confirmed MHC associations in human studies as well as revealing further associations and the importance of epistasis [32].

In early genetic studies of canine hypoadrenocorticism, microsatellite markers in the vicinity of *CTLA4* were shown to be associated with hypoadrenocorticism in Portuguese Water Dogs (PWDs) and Nova Scotia duck tolling retrievers [24, 33]. However, when the sequence around *CTLA4* was analysed in PWDs, three haplotypes were identified that did not segregate convincingly with hypoadrenocorticism, although further details were not provided [24]. More recent candidate gene studies, undertaken across several breeds, have revealed a SNP in the CTLA4 promoter region, to be associated with hypoadrenocorticism in springer spaniels but not in other breeds [34, 35]. Although there are no reported SNPs in the canine *CTLA4* coding sequence, the promoter region has been characterised in some detail [36, 37], revealing 20 SNPs and three indels within the space of 1.6 kB upstream of the start codon. These variants segregate into 17 distinct haplotypes. Significant allele and haplotype associations have been reported between the *CTLA4* promoter region and diabetes [36], although not for immune-mediated haemolytic anaemia [37]. This high degree of polymorphism in the canine *CTLA4* promotor has the potential to impact on protein expression, which may, in turn, influence its immune regulatory function and susceptibility to autoimmunity. Our hypothesis is that CTLA4 plays a role in the immunopathogenesis of canine hypoadrenocorticism and the study aimed to examine *CTLA4* promotor alleles and haplotypes in selected breeds.

## Materials and methods

Samples consisted of the residual volume of blood samples following completion of diagnostic testing at the Royal Veterinary College (RVC) Diagnostic Laboratories (Hertfordshire, UK) or NationWide Laboratories (NWL) (Poulton-le-Fylde, UK); blood samples were not specifically taken for this study in line with the ethical approval for the project and United Kingdom law. Furthermore, due to the nature of the ethical approval for this project (i.e. submitted samples were required to be de-identified) and to comply with UK data protection regulations, it was not possible to explore the relatedness of the animals, whose DNA samples were genotyped in the study. Further genomic DNA (gDNA) samples from dogs affected by hypoadrenocorticism were provided from the UK DNA Archive for Companion Animals (Universities of Liverpool and Manchester). A cohort of affected dogs comprised the populations described in previous genetic analyses in hypoadrenocorticism, [34, 35]. The control (unaffected) dogs are unique to this paper and were accessed from the RVC Genetic Archive. The cocker spaniels affected with hypoadrenocorticism are common to both studies; ten affected springer spaniels are unique to this study and four affected WHWT are unique to this study.

Dogs affected with hypoadrenocorticism were identified from clinical and diagnostic records with pre- and post- ACTH stimulation test results consistent with corticosteroid deficiency (both < 27.6 nmol/L) and no known history of recent steroid administration. Genomic DNA from breed matched control dogs over 9 years old with no known history of endocrinopathy or immune-mediated disease were selected from the RVC Genetic Archive. All samples were handled and processed according to local laboratory protocols and procedures until they entered the study at which point they were stored at − 20 °C in RNase, DNase, DNA and PCR-inhibitor free polypropylene tubes. Transfer of samples between locations was performed frozen on dry ice.

The RVC has ethical approval for residual clinical material, taken for diagnostic purposes, to be used for research with informed owner consent. NationWide Laboratories has approval for utilising clinical material for development of diagnostic assays, provided that anonymity is maintained and data protection is observed.

### Extraction of nucleic acid and PCR

**Fig. 1:**
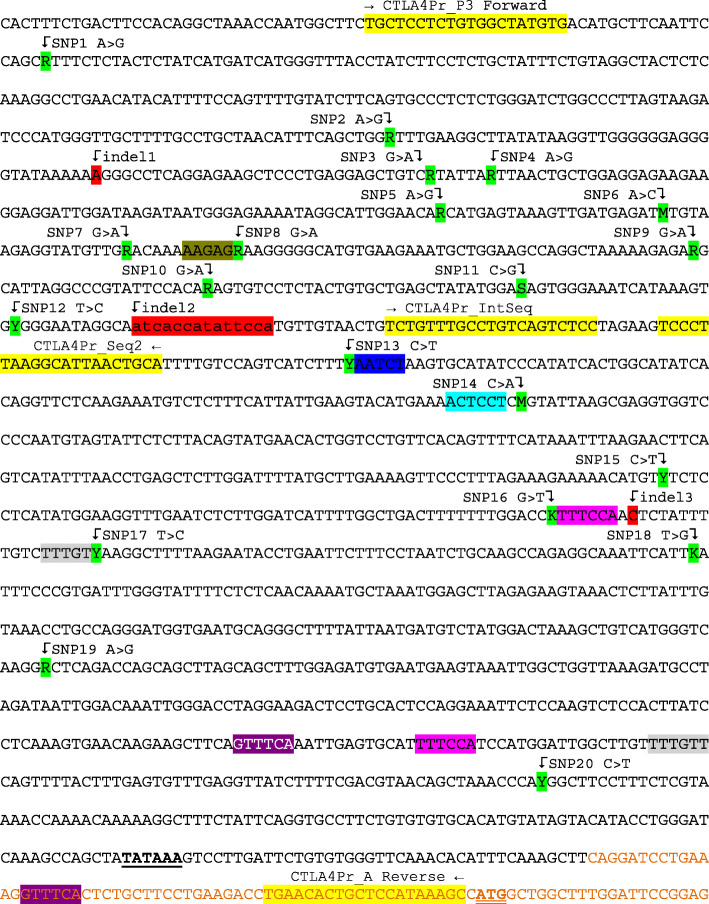
Canine CTLA4 promoter region variation and selected response elements. *Primer binding sites are shown, highlighted yellow with arrow denoting the starting base in a 5′-3′ direction. Variations are as named, indicated by**or**; SNPs highlighted green, Dog Genome Assembly allele followed by variant shown; INDELs highlighted red, capital letters denote a variant present in, and lowercase letters denote variation not present in, the dog genome assembly sequence. Predicted response elements NFAT TTTCC (a) highlighted pink; partial SP1 site highlighted turquoise; partial GATA1 site highlighted blue; predicted AP-1 site highlighted purple; FoxP3 TTTGTT highlighted grey; TCF site highlighted dark green. SNP codes: M: A/C; R: A/G; S: C/G; Y: C/T; K: G/T. Exon sequence is orange. The ATG start codon isbold double underlined.The TATA box isbold single underlined*

Genomic DNA extraction and subsequent PCR was performed as previously described [37]. Briefly, gDNA was extracted from EDTA blood using the GenElute Blood Genomic Extraction Kit (Sigma-Aldrich, UK) according to the manufacturer’s instructions. Polymerase chain reaction (PCR) was used to amplify the *CTLA4* promoter region (1.6 kb upstream of exon 1) using custom designed primers (CTLA4 promoter sense: 5′-TGCTCCTCTGTGGCTATGTG-3′ and CTLA4 promoter antisense: 5′-TGAACACTGCTCCATAAAGC-3′) (Fig. 1). PCR was performed in 50 μL reactions, containing 2 μL gDNA as template and 4 μL primer mix (20 pmol/μL total concentration CTLA4 promoter-specific primers). Each reaction also contained 10 μL Hi-Spec® additive, 5 μL 10x ImmunoBuffer®, 2.5 μL MgCl_2_ (2.5 mM final concentration), 0.5 μL dNTP (1 mM final concentration) and 0.2 μL Immolase® DNA polymerase (2.5 IU) (all Bioline). Reactions were heated to 95 °C for 10 min (polymerase activation), followed by 35 cycles consisting of 94 °C for 40 s (denaturation), 60 °C for 30 s (annealing) and 72 °C for 2 min (elongation); with a final extension step at 72 °C for 10 min. The reactions were performed using a G-Storm® GS1 Thermal Cycler (Gene Technologies Ltd., Essex, UK)**.**

PCR products were separated by horizontal gel electrophoresis in 2% agarose dissolved in Tris/borate/EDTA (TBE) buffer (both from Bioline) containing 1.5 μL/mL SafeView™ Nucleic Acid Stain (NBS Biologicals Ltd., Huntingdon, UK) and visualised under 590 nm UV light (ImageMaster VDS®, Pharmacia Biotech/GE Healthcare, Buckinghamshire, UK). Amplicons were excised and DNA extracted using the GenElute Gel Extraction Kit (Sigma-Aldrich, UK) according to the manufacturer’s instructions. PCR products were submitted for Sanger sequencing (Source Bioscience, UK) using the PCR primers and two additional primers for internal sequencing (CTLA4 Promoter Sequencing 5′-TCTGTTTGCCTGTCAGTCTCC-3′ and CTLA4 promoter Sequencing 2 5′-TGCAGTTAATGCCTTAAGGGA-3′). Chromatograms were analysed using BioEdit Sequence Alignment Editor Software. Previously documented SNPs and indels were analysed and haplotypes assigned for each dog (Table 1) [37]. The call rate for all markers was 100%, achieved through repeat PCR and sequencing when necessary.

**Table 1:**
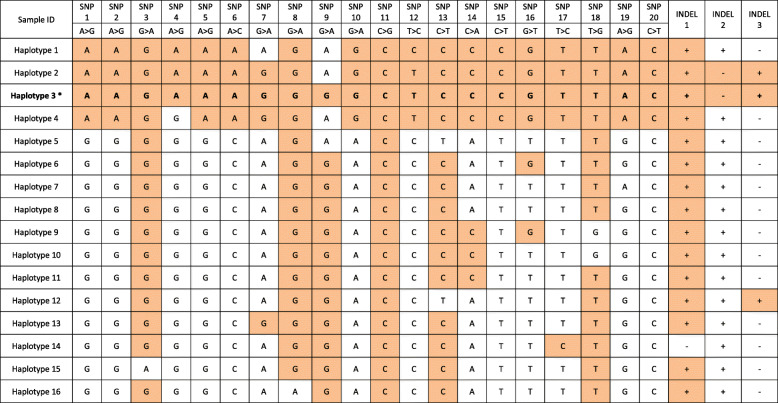
*CTLA4 promoter haplotypes.*Haplotype 3 (*) is the reference haplotype derived from the dog genome assembly sequence and dog genome assembly SNPs are in shaded cells

Data were organised in Microsoft® Excel© 2013 version 15 (Microsoft Corporation, Redmond, WA, USA) and statistical analyses performed using PLINK version 1.07^1^ (Shaun Purcell) and IBM© SPSS© Statistics for Windows, Version 24.0 (IBM Corp, Armonk, NY, USA). An exact method was used for the testing of Hardy Weinberg equilibrium, due to the presence of rare genotypes [38]. Categorical data were analysed using contingency tables, with Fisher’s exact test used for comparisons. Permutation testing (10,000 permutations using the max (T) method in PLINK) was used to correct for multiple testing when individual markers were assessed. The cut off for significance was set at *p* < 0.05.

## Results

Samples from springer spaniels (*n* = 28 cases, *n* = 57 controls), cocker spaniels (*n* = 19 cases, *n* = 51 controls) and WHWT (*n* = 28 cases and *n* = 31 controls) were included in the study.

Sex and age information was only available for 10 springer spaniel cases, seven female and three male, which was not significantly different to the control population (*p* = 0.168) comprising of 24 female and 33 male dogs. A significant age difference was found in springer spaniels (*p* < 0.001) with controls (572; 468–728 wk) older than cases (364; 156–520 wk). The medical conditions for which the control population presented included 15 dogs with a neurological condition, including seven with intervertebral disc disease (IVDD) and three with epilepsy, four dogs had an orthopaedic complaint, four infections, two each with neoplasia, cardiac disease, chronic renal failure and a toxic insult. Several dogs had more than one concurrent medical complaint.

Sex and age information was only available for 12 of the cocker spaniel cases, ten female and two male, which was significantly different to the control population (*p* = 0.049) comprised of 23 female and 35 male dogs. A significant age difference was found found in the cocker spaniels (*p* = 0.001) with controls (520; 468–728 wk) older than cases (286; 156–598 wk). The medical conditions for which the control population presented included 20 dogs with neoplasia, 12 with IVDD, six with cardiac disease, two each with epilepsy and orthopaedic disease. Several dogs had more than one concurrent medical complaint.

Sex and age information was only available for 14 of the WHWT cases, 10 female and four male which was not significantly different to the control population (*p* = 0.051) comprised of 11 female and 20 male dogs. A significant age difference was found between the WHWT groups (*p* < 0.001) with controls (588; 486 – 780wk) older than cases (217; 13–464 wk). The medical conditions for which the control population presented included nine dogs with respiratory disease, of which seven had idiopathic pulmonary fibrosis, five dogs with renal disease, four with orthopaedic disease, four with neurological disease and two each with oesophageal foreign bodies and trauma. Several dogs had more than one concurrent medical complaint.

Minor allele frequencies are shown in Table 2, showing some degree of disparity between the three breeds. All variants were in Hardy Weinberg equilibrium for case, control and whole populations for cocker spaniels and springer spaniels. For WHWT, 13/23 variants were not in equilibrium for the case population, whilst being in equilibrium within the control population (Table 3); due to this the WHWT haplotypes and nucleotide variants were not assessed further.

**Table 2:** Minor allele frequencies*. SNPs and indels are shown in 5’ to 3’ order from top to bottom*

Marker	WHWT		Springer Spaniel		Cocker Spaniel	
	Minor Allele	MAF	Minor Allele	MAF	Minor Allele	MAF
SNP1	A	0.102	A	0.38	A	0.086
SNP2	A	0.102	A	0.38	A	0.086
SNP3	A	0	A	0.0059	A	0.0071
SNP4	A	0.102	A	0.38	A	0.086
SNP5	A	0.102	A	0.38	A	0.086
SNP6	A	0.102	A	0.38	A	0.086
SNP7	G	0.102	G	0.39	G	0.086
SNP8	A	0	A	0.012	A	0
SNP9	A	0.102	A	0.36	A	0.014
SNP10	G	0.102	G	0.38	G	0.086
SNP11	G	0	G	0	G	0
SNP12	T	0.102	T	0.38	T	0.086
SNP13	T	0.034	T	0.14	T	0.036
SNP14	A	0.49	C	0.4	C	0.093
SNP15	C	0.102	C	0.38	C	0.086
SNP16	G	0.102	G	0.38	G	0.086
SNP17	C	0	C	0	C	0
SNP18	G	0.407	G	0.0059	G	0
SNP19	A	0.102	A	0.38	A	0.086
SNP20	T	0	T	0	T	0
indel1	–	0	–	0	–	0
indel2	–	0.102	–	0.38	–	0.086
indel3	+	0.14	–	0.49	+	0.12

**Table 3:** Hardy Weinberg Equilibrium in WHWTs *SNPs and indels are shown in 5’ to 3’ order top to bottom; 118 WHWT alleles counted for each marker with 100% call rate. Genotype frequencies are shown as homozygote for minor allele/heterozygote/homozygote for major allele. For significant deviation from Hardy Weinberg equilibrium the p value is highlighted in red*

Marker	Minor Allele	Group	Genotype frequency	Observed Heterozygotes	Expected Heterozygotes	*P* value
SNP1	A	Case	2–1 - 25	0.036	0.163	0.005
		Control	0–7 - 24	0.226	0.2	1
SNP2	A	Case	2–1 - 25	0.036	0.163	0.005
		Control	0–7 - 24	0.226	0.2	1
SNP3	A	Case	0–0 - 28	0	0	1
		Control	0–0 - 31	0	0	1
SNP4	A	Case	2–1 - 25	0.036	0.163	0.005
		Control	0–7 - 24	0.226	0.2	1
SNP5	A	Case	2–1 - 25	0.036	0.163	0.005
		Control	0–7 - 24	0.226	0.2	1
SNP6	A	Case	2–1 - 25	0.036	0.163	0.005
		Control	0–7 - 24	0.226	0.2	1
SNP7	G	Case	2–1 - 25	0.036	0.163	0.005
		Control	0–7 - 24	0.226	0.2	1
SNP8	A	Case	0–0 - 28	0	0	1
		Control	0–0 - 31	0	0	1
SNP9	A	Case	2–1 - 25	0.036	0.163	0.005
		Control	0–7 - 24	0.226	0.2	1
SNP10	G	Case	2–1 - 25	0.036	0.163	0.005
		Control	0–7 - 24	0.226	0.2	1
SNP11	G	Case	0–0 - 28	0	0	1
		Control	0–0 - 31	0	0	1
SNP12	T	Case	2–1 - 25	0.036	0.163	0.005
		Control	0–7 - 24	0.226	0.2	1
SNP13	T	Case	0–3 - 25	0.107	0.101	1
		Control	0–1 - 30	0.032	0.032	1
SNP14	A	Case	7–11 - 10	0.393	0.494	0.275
		Control	9–15 - 7	0.484	0.498	1
SNP15	C	Case	2–1 - 25	0.036	0.163	0.005
		Control	0–7 - 24	0.226	0.2	1
SNP16	G	Case	2–1 - 25	0.036	0.163	0.005
		Control	0–7 - 24	0.226	0.2	1
SNP17	C	Case	0–0 - 28	0	0	1
		Control	0–0 - 31	0	0	1
SNP18	G	Case	7–12 - 9	0.429	0.497	0.466
		Control	5–12 - 14	0.387	0.458	0.438
SNP19	A	Case	2–1 - 25	0.036	0.163	0.005
		Control	0–7 - 24	0.226	0.2	1
SNP20	T	Case	0–0 - 28	0	0	1
		Control	0–0 - 31	0	0	1
indel1	–	Case	0–0 - 28	0	0	1
		Control	0–0 - 31	0	0	1
indel2	–	Case	2–1 - 25	0.036	0.163	0.005
		Control	0–7 - 24	0.226	0.2	1
indel3	+	Case	2–4 - 22	0.143	0.245	0.066
		Control	0–8 - 23	0.258	0.225	1

### Genotype and haplotype associations

Testing for association of genotype (*p* = 0.669) and haplotype (*p* = 0.291) with hypoadrenocorticism in springer spaniels did not reveal any overall significant associations, although haplotype 2 was overrepresented in post-hoc testing (Table 4). No effect of homozygosity was found (*p* = 0.474). For cocker spaniels, there were significant associations between disease phenotype and *CTLA4* promoter genotype (*p* = 0.006) with dogs homozygous for haplotype 8 more likely in controls (43/51) than cases (10/19) (*p* = 0.0034) no other significant genotype associations were found. Cocker spaniel also had significant associations with haplotype (p = 0.003) with haplotypes 3 (OR = 4.59, *p* = 0.0247) and 12 (OR = 11.8, *p* = 0.0193) associated with increased odds of hypoadrenocorticism and haplotype 8 (OR = 0.20, *p* = 0.0038) associated with decreased odds of hypoadrenocorticism (Table 5). Cases were more likely to be heterozygous (*p* = 0.011).

**Table 4:** *CTLA4* promoter haplotypes present in springer spaniels. *Number of each haplotype is shown (with frequency in brackets) for cases, 2n = 56, and controls, 2n = 114.* NaN: not a number; OR: odds ratio; 95% CI: 95% confidence intervals of the OR. *p values for individual 2 × 2 tables are given with significant results* coloured red, overall significance *p* = 0.291

*CTLA4* promoter Haplotype	Cases 2n (%)	Controls 2n (%)	p	OR 95% CI
2	27 (48.2%)	34 (29.8%)	0.0266	2.19 (1.13–4.23)
3	1 (1.78%)	2 (1.75%)	1	1.01 (0.09–11.4)
8	21 (37.5%)	57 (50%)	0.1423	0.6 (0.31–1.15)
10	0 (0%)	1 (0.87%)	1	0
11	0 (0%)	3 (2.63%)	0.5516	0
12	7 (12.5%)	16 (14.0%)	1	0.87 (0.33–2.26)
15	0 (0%)	1 (0.87%)	1	0

**Table 5:** *CTLA4* promoter haplotypes present in cocker spaniels. *Number of each haplotype is shown (with frequency in brackets) for cases, 2n = 38, and controls, 2n = 102.* NaN: not a number; OR: odds ratio; 95% CI: 95% confidence intervals of the OR. *p values for individual 2 × 2 tables are given with significant results* coloured red, overall significance *p* = 0.003

*CTLA4* promoter Haplotype	Cases 2n (%)	Controls 2n (%)	p	OR (95% CI)
2	0 (0%)	2 (1.96%)	1.0000	0
**3**	6 (15.7%)	4 (3.92%)	0.0247	4.59 (1.21–17.3)
**8**	27 (71.0%)	94 (92.1%)	0.0038	0.20 (0.07–0.57)
11	1 (2.63%)	0	0.2714	NaN
**12**	4 (10.5%)	1 (0.98%)	0.0193	11.8 (1.28–110)
15	0 (0%)	1 (0.98%)	1.0000	0

### Specific allele associations

To further elucidate the role of specific polymorphisms within associated haplotypes in cocker spaniels and to determine whether any polymorphisms segregated with hypoadrenocorticism outside full haplotypes in springer spaniels, SNPs and indels were analysed individually. In springer spaniels 14/23 markers segregated with disease, 11/14 were in linkage disequilibrium and the minor alleles were associated with hypoadrenocorticism (OR 2.16, *p* = 0.026). Of the further 3/14 markers, for SNP7 (OR = 2.5, *p* = 0.008) and SNP9 (OR = 2.19, *p* = 0.027), the minor allele was associated with hypoadrenocorticism and for indel3, the minor allele was associated with decreased odds of hypoadrenocorticism (OR = 0.5, *p* = 0.031) (Table 6). Three markers segregated with hypoadrenocorticism in the cocker spaniels, with the minor allele in SNP13 (OR = 11.88, *p* = 0.016), SNP14 (OR = 3.61, *p* = 0.031) and indel3 (OR = 4.43, *p* = 0.006), associated with an increased risk of hypoadrenocorticism (Table 7).

**Table 6:** Association of *CTLA4* polymorphisms with hypoadrenocorticism in springer spaniels. *Markers are as* Table *3**, 5′ to 3′ order from top to bottom SNPs and then indels. MAF: minor allele frequency; NaN: not a number; N/A: not calculable; OR: odds ratio; 95% CI: 95% confidence intervals of the OR; Max (T) p value calculated by permutation analysis; FDR: false discovery rate*

Marker	Minor allele	MAF in cases	MAF in controls	Max (T) *p* value	FDR	OR (95% CI)
SNP1	A	0.5	0.315	0.026	0.03462	2.16 (1.12–4.17)
SNP2	A	0.5	0.315	0.026	0.03462	2.16 (1.12–4.17)
SNP3	A	0	0.008	1	1	0 (0 - NaN)
SNP4	A	0.5	0.315	0.026	0.03462	2.16 (1.12–4.17)
SNP5	A	0.5	0.315	0.026	0.03462	2.16 (1.12–4.17)
SNP6	A	0.5	0.315	0.026	0.03462	2.16 (1.12–4.17)
SNP7	G	0.535	0.315	0.008	0.03462	2.5 (1.29–4.82)
SNP8	A	0.035	0	0.132	0.1072	N/A
SNP9	A	0.482	0.298	0.027	0.03462	2.19 (1.13–4.23)
SNP10	G	0.5	0.315	0.026	0.03462	2.16 (1.12–4.17)
SNP11	N/A	0	0	1	1	N/A
SNP12	T	0.5	0.315	0.026	0.03462	2.16 (1.12–4.17)
SNP13	T	0.125	0.140	1	1	0.87 (0.33–2.26)
SNP14	C	0.5	0.350	0.071	0.07347	1.85 (0.96–3.54)
SNP15	C	0.5	0.315	0.026	0.03462	2.16 (1.12–4.17)
SNP16	G	0.5	0.315	0.026	0.03462	2.16 (1.12–4.17)
SNP17	N/A	0	0	1	1	N/A
SNP18	G	0	0.008	1	1	0 (0 - NaN)
SNP19	A	0.5	0.315	0.026	0.03462	2.16 (1.12–4.17)
SNP20	N/A	0	0	1	1	N/A
indel1	N/A	0	0	1	1	N/A
indel2	–	0.5	0.315	0.026	0.03462	2.16 (1.12–4.17)
indel3	–	0.375	0.543	0.031	0.0571	0.50 (0.26–0.96)

**Table 7:** Association of *CTLA4* polymorphisms with hypoadrenocorticism in cocker spaniels. *Markers are as Table 1, 5′ to 3′ SNPs then indels top to bottom. MAF: minor allele frequency; NaN: not a number; N/A: not calculable; OR: odds ratio; 95% CI: 95% confidence intervals of the OR. Max (T) p value calculated by permutation analysis; FDR: false discovery rate*

Marker	Minor allele	MAF in cases	MAF in controls	Max (T) *p* value	FDR	OR (95% CI)
SNP1	A	0.158	0.059	0.082	0.09887	3 (0.9–9.9)
SNP2	A	0.158	0.059	0.082	0.09887	3 (0.9–9.9)
SNP3	A	0	0.0098	1	1	0 (0 - NaN)
SNP4	A	0.158	0.059	0.082	0.09887	3 (0.9–9.9)
SNP5	A	0.158	0.059	0.082	0.09887	3 (0.9–9.9)
SNP6	A	0.158	0.059	0.082	0.09887	3 (0.9–9.9)
SNP7	G	0.158	0.059	0.082	0.09887	3 (0.9–9.9)
SNP8	N/A	0	0	1	1	N/A
SNP9	A	0	0.01961	1	1	0 (0 - NaN)
SNP10	G	0.158	0.059	0.082	0.09887	3 (0.9–9.9)
SNP11	N/A	0	0	1	1	N/A
SNP12	T	0.158	0.059	0.082	0.09887	3 (0.9–9.9)
SNP13	T	0.105	0.0098	0.016	0.09887	11.88 (1.2–110)
SNP14	C	0.184	0.059	0.031	0.09887	3.61 (1.1–11.5)
SNP15	C	0.158	0.059	0.082	0.09887	3 (0.9–9.9)
SNP16	G	0.158	0.059	0.082	0.09887	3 (0.9–9.9)
SNP17	N/A	0	0	1	1	N/A
SNP18	N/A	0	0	1	1	N/A
SNP19	A	0.158	0.059	0.082	0.09887	3 (0.9–9.9)
SNP20	N/A	0	0	1	1	N/A
indel1	N/A	0	0	1	1	N/A
indel2	–	0.158	0.059	0.082	0.09887	3 (0.9–9.9)
indel3	+	0.263	0.067	0.0097	0.05783	4.85 (1.6–13.9)

## Discussion

Within canine hypoadrenocorticism, two previous studies analysed genetic variants in the region of the CTLA4 locus [24, 33] and two studies focussed on a single CTLA4 promoter SNP as a susceptibility marker (SNP 16 in this study) [34, 35]. This study was therefore designed to conduct a more detailed analysis of variation in the CTLA4 promoter region in dogs with hypoadrenocorticism.

Two populations of dog with hypoadrenocorticism in this study, cocker spaniels and springer spaniels, overlap with previous analyses [34, 35]. However, this study has expanded case populations, novel control populations and has extended the analysis from a single SNP to a wider, previously described, promoter region [37]. The control populations used in this study were selected based on a more stringent phenotyping compared to prior studies [34, 35]. Despite a more rigorous inclusion/exclusion criteria, the phenotyping and selection of unaffected dogs was still limited by the information available from the available clinical records. It is possible that dogs might have had historical or concurrent disease(s) not recorded in the electronic patient record. Incorrect phenotyping in this study would lead to the erroneous inclusion of individuals with immune-mediated conditions; which might decrease the power of this study to identify a difference rather than bias towards a type one error. In this study the case phenotyping is robust given the nature of the diagnostic criteria; however, any incorrect phenotyping would also bias towards a type two error.

Permutation analysis was used to correct for familywise error in this study as well as false discovery rate correction. Given the analysis of a single locus with several highly linked markers, this choice seems appropriate and caution must be used in interpreting the results.

Genotyping errors from low call rates and missing samples can limit or bias genetic analyses with these effects exaggerated in candidate gene studies with relatively small numbers [39]. Every effort was made to identify and call each polymorphism in this study leading to a 100% call rate eliminating this as a source of bias.

The WHWT case population selected for this study was found not to be in HWE. Inferences of significant associations with disease can made regarding deviation from HWE in case but not control populations [40, 41]. However, deviation from HWE can also be caused by a number of factors, including genotyping errors, population stratification and sampling methods. Sampling in this study was performed through two centres, therefore population stratification is possible, with local genetic pools of dogs contributing to these differences. As outlined in the methods, no further exploration of relatedness of dogs was possible. These data should therefore be interpreted with caution, both due to the small sample size and possible stratification issues [40]. It remains possible that the loss of HWE in the case population reflects an association of the SNPs and indel2 with hypoadrenocorticism.

Springer spaniels were not found to have significant associations between hypoadrenocorticism and genotype, heterozygosity or haplotypes. Significant associations were found with several variants, primarily with SNPs making up haplotype 2 which was overrepresented in post-hoc haplotype analysis (Table 4). Haplotype 2 only differs from haplotype 3, overrepresented in the closely related cocker spaniels with hypoadrenocorticism, by a G to A substitution at SNP6 (Table 1). The potential significance of possibly biologically relevant individual variants is discussed later.

In cocker spaniels, a homozygous 8/8 genotype was found to be associated with an absence of hypoadrenocorticism. Following this, haplotypes 3 and 12 were both identified as risk haplotypes and haplotype 8 as a protective haplotype with affected dogs more likely to be heterozygotic. Haplotype 3 was not identified as a risk haplotype for diabetes mellitus in a previous study [36]. However, the closely related haplotype 2 was associated with diabetes mellitus in Border terriers with an opposite association in West Highland white terriers. Haplotype 12 has not been identified as a risk haplotype previously but reported as protective for diabetes in Samoyed and miniature Schnauzers, and neutral in WHWT and crossbreed dogs. Haplotype 8, a protective haplotype for hypoadrenocorticism in cocker spaniels, has also been reported as protective for diabetes in Border terriers; however, it was neutral in Labradors, miniature Schnauzers, West Highland white terriers and crossbreed dogs and associated with an increased risk of diabetes in Samoyeds.

Across eight breeds and three diseases, the same SNPs and haplotypes have been variously described as conferring risk, being protective or neutral [36, 37]. There are a number of possible reasons for these findings. Firstly, these risks may be disease specific with hypoadrenocorticism risks changing differently to those for IMHA or diabetes or the gene may or may not be involved in the aetiology and pathogenesis of any specific disease.

The same haplotype could have a different relative risk profile between different breeds; for example, if haplotype 3 is relatively protective to haplotype 8 and haplotype 8 is relatively protective to haplotype 12, then haplotype 8 will appear as either increasing or decreasing risk depending on the other haplotypes present in the breed and in what proportions. Breed specific genetic backgrounds might also influence the effect of *CTLA4* promoter variation, due to epistasis. A specific example of epistasis and CTLA4 is known in humans, between CTLA4 and TNF-alpha. A specific TNF–alpha variant is only associated with an altered risk of primary biliary cirrhosis when there is an AA genotype, not AG or GG, at the − 318 CTLA4 promoter polymorphism [42]. Breed differences may be similar to those seen in different ethnic groups in the human population, e.g. different CTLA4 3’UTR (AT) n associations are apparent in Japanese and Portuguese patients with SLE [43], or different associations of CTLA4 polymorphisms between distinct populations with Addison’s disease [18]. The interactions between breed, specific epistasis and disease are likely to be complex and require deeper genetic analysis in large cohorts of well phenotyped dogs to understand.

Although transcription factor-binding sites are not fully conserved between species [44] cross-species comparisons can be used to identify potential regulatory sites [45], possibly due to conserved core binding motifs [46]. There has been little research undertaken on the nature of canine response elements and transcription factor binding characteristics, therefore interpretation of effects of SNPs on binding sites was undertaken based upon conserved aspects of promoter biology sourced from the JASPAR database using the online Transcription Element Search System (TESS; www.cbil.upenn.edu/tess) and literature searches. Since computational methods used alone can deliver high false positive rates [47, 48] below we have focussed on selected sites of potential biological significance.

Similar to the findings for springer spaniels and hypoadrenocorticism in this study, SNP13T has previously been associated with increased risk of diabetes in Labradors [34]; however, this variant was also associated with a decreased diabetes risk in miniature Schnauzers and Samoyeds. SNP13 is located at the start of a GATA-1 binding site [49]. GATA-1 has been shown to up-regulate *CTLA4* transcription and to be able to initiate a substantial component of the Treg transcriptional signature, when expressed in conjunction with FoxP3 in non-regulatory murine T cells [50]. *CTLA4* promoter SNP14 has not previously been associated with disease risk, however unusually it was found to change minor allele status between breeds [36]. SNP14 lies adjacent to a core SP1 promoter site. A polymorphism in the promoter region of ICOS in humans is associated with an SP1 site [51] and SP1 is known to affect *CIITA* transcription in dendritic cells [52]. SP1 also appears to bind upstream of *Foxp3* in nTregs, which indicates its potential importance in immune responses. SP1 binding can protect CpG sites from methylation [53] and the SNP14A polymorphism removes a CpG motif from the canine *CTLA4* promoter.

This is the first association between the *CTLA4* promoter indel3 and canine disease, with the presence of a C at indel3 being the most significantly associated risk marker for hypoadrenocorticism in cocker spaniels (*p* = 0.0097) and springer spaniels (*p* = 0.031). Indel3 is situated between a potential NFAT binding site [54] and a putative FoxP3 site [55], which are separated by 12 or 13 bases depending on the indel3 status (Fig. 1). NFAT typically binds to DNA with a partner, e.g. FoxP3 [56] and both FoxP3 and NFAT response elements are also located close together in the *CTLA4* promoter in humans [55, 57]. In mice, FoxP3 and NFAT co-operate in up-regulating CTLA4 expression and disrupting interaction between these two transcription factors impairs CTLA4 transcription, impacting on Treg function [58]. Taking into consideration previous findings in other species, the association of SNP16G with canine diabetes mellitus and hypoadrenocorticism and the association of the C insertion with hypoadrenocorticism in cocker spaniels and springer spaniels, this small region of the canine *CTLA4* promoter is very interesting for further investigation.

SNP7, associated with hypoadrenocorticism in springer spaniels, is adjacent to a predicted binding site for T cell factor 1 (TCF-1), one of a family of TCF/Lymphoid enhancer-binding factor (LEF) transcription factors forming part of the wnt signalling pathway [59]. TCF is particularly important during thymic development [60], which might be mediated by wnt3a [61]. The wnt signalling pathway, including TCF-1 and LEF-1, can also modulate mature T cell function, including prolonging regulatory T cell (Treg) survival and therefore, potentially impacting on autoimmunity [61]. Four potential TCF/LEF sites have been identified within the 800 bp upstream of the start codon in human *CTLA4* [62], one of which contains the C(− 318) T SNP [63] associated with altered promoter activity [63, 64] and with susceptibility to Grave’s disease [65], autoimmune pancreatitis [66] but not diabetes [67, 68]. In one study, when a melanoma cell line was treated with wnt3a, *CTLA4* was the most up-regulated gene, which was proposed to represent a mechanism of immune evasion [62]. In Jurkat and HeLa cell lines, addition of LEF-1 has been shown to increased *CTLA4* promoter activity, with the -318 T variant consistently more active than the -318C variant [63].

It is highly likely that some of the promoter variants identified do not confer any functional consequences for expression of canine CTLA4. In mice, seven intronic SNPs and three intronic indels have been found, but these were not associated with any functional outcomes [69]. Also, any polymorphisms that segregate with hypoadrenocorticism could be merely in linkage disequilibrium with enhancers, silencers, insulators or other locus control regions [70]. In a large study of the human *CTLA4* region, 108 SNPs were typed, 78 of which were in a 100 kb *CTLA4* LD block, only 23 of these were not significantly associated with Graves’ disease at *p* < 0.05 and mathematical modelling was used to determine which variation was most likely functionally associated with disease [71]. Given that LD blocks in dogs are larger than in human beings [72] the possibility of genes in this region (especially ICOS or CD28) being involved remains, although a microsatellite marker flanking canine CD28 was not associated with hypoadrenocorticism in NSDTRs in one study [33].

## Conclusions

This study of canine *CTLA4* promoter variants in three dog breeds adds further detail to previous research and provides some evidence that *CTLA4* may from part of the susceptibility aetiology of canine hypoadrenocorticism in some breeds. This work supports the belief that the pathophysiology of hypoadrenocorticism is immune-mediated. This study represents a further step in unravelling the complex genetics of hypoadrenocorticism and in establishing a potential role for *CTLA4.*

## Data Availability

All data generated or analysed during this study are included in this published article.

## Footnotes

1 http://pngu.mgh.harvard.edu/purcell/plink/

## References

[1] Scott-Moncrieff JC. Hypoadrenocorticism. In: Canine Feline Endocrinol: Elsevier; 2015. p. 485–520. 10.1016/B978-1-4557-4456-5.00012-2.

[2] Frank CB, Valentin SY, Scott-Moncrieff JCR, Miller MA (2013). Correlation of inflammation with adrenocortical atrophy in canine Adrenalitis. J Comp Pathol.

[3] Chase K, Lawler DF, McGill LD, Miller S, Nielsen M, Lark KG (2010). Age relationships of postmortem observations in Portuguese water dogs. Age (Omaha).

[4] Zelissen PMJ, Bast EJEG, Croughs RJM (1995). Associated autoimmunity in Addison’s disease. J Autoimmun.

[5] Boag AM, Christie MR, McLaughlin KA, Syme HM, Graham PA, Catchpole B (2015). Autoantibodies against cytochrome P450 side-chain cleavage enzyme in dogs (Canis lupus familiaris) affected with Hypoadrenocorticism (Addison’s disease). PLoS One.

[6] Boag AM, Christie MR, McLaughlin KA, Syme HM, Graham P, Catchpole B (2018). A longitudinal study of autoantibodies against cytochrome P450 side-chain cleavage enzyme in dogs (Canis lupus familiaris) affected with hypoadrenocorticism (Addison’s disease). Vet Immunol Immunopathol.

[7] Forabosco P, Bouzigon E, Ng MY, Hermanowski J, Fisher SA, Criswell LA (2008). Meta-analysis of genome-wide linkage studies across autoimmune diseases. Eur J Hum Genet.

[8] Husebye E, Løvås K (2009). Pathogenesis of primary adrenal insufficiency. Best Pract Res Clin Endocrinol Metab.

[9] Criswell LA, Pfeiffer KA, Lum RF, Gonzales B, Novitzke J, Kern M (2005). Analysis of families in the multiple autoimmune disease genetics consortium (MADGC) collection: the PTPN22 620W allele associates with multiple autoimmune phenotypes. Am J Hum Genet.

[10] Gough SC, Walker LS, Sansom DM (2005). CTLA4 gene polymorphism and autoimmunity. Immunol Rev.

[11] Pearce SH, Merriman TR (2006). Genetic progress towards the molecular basis of autoimmunity. Trends Mol Med.

[12] Vaidya B, Pearce S (2004). The emerging role of the CTLA-4 gene in autoimmune endocrinopathies. Eur J Endocrinol.

[13] Kristiansen OP, Larsen ZM, Pociot F (2000). CTLA-4 in autoimmune diseases a general susceptibility gene to autoimmunity. Genes Immun.

[14] Kemp EH, Ajjan RA, Husebye ES, Peterson P, Uibo R, Imrie H (1998). A cytotoxic T lymphocyte antigen-4 (CTLA-4) gene polymorphism is associated with autoimmune Addison’s disease in English patients. Clin Endocrinol.

[15] Donner H, Braun J, Seidl C, Rau H, Finke R, Ventz M (1997). Codon 17 polymorphism of the cytotoxic T lymphocyte antigen 4 gene in Hashimoto’s thyroiditis and Addison’s disease. J Clin Endocrinol Metab.

[16] Vaidya B, Imrie H, Geatch DR, Perros P, Ball SG, Baylis PH (2000). Association analysis of the cytotoxic T lymphocyte antigen-4 (CTLA-4) and autoimmune regulator-1 (AIRE-1) genes in sporadic autoimmune Addison’s disease. J Clin Endocrinol Metab.

[17] Brozzetti A, Marzotti S, Tortoioli C, Bini V, Giordano R, Dotta F (2010). Cytotoxic T lymphocyte antigen-4 Ala17 polymorphism is a genetic marker of autoimmune adrenal insufficiency: Italian association study and meta-analysis of European studies. Eur J Endocrinol.

[18] Wolff ASB, Mitchell AL, Cordell HJ, Short A, Skinningsrud B, Ollier W (2015). CTLA-4 as a genetic determinant in autoimmune Addison’s disease. Genes Immun.

[19] Pérez de Nanclares G, Martín-Pagola A, Ramón Bilbao J, Vázquez F, Castaño L (2004). No evidence of association of CTLA4 polymorphisms with Addison’s disease. Autoimmunity.

[20] Falorni A, Brozzetti A, Perniola R (2016). From genetic predisposition to molecular mechanisms of autoimmune primary adrenal insufficiency. Front Horm Res.

[21] Oberbauer AM, Benemann KS, Belanger JM, Wagner DR, Ward JH, Famula TR (2002). Inheritance of hypoadrenocorticism in bearded collies. Am J Vet Res.

[22] Famula TR, Belanger JM, Oberbauer AM (2003). Heritability and complex segregation analysis of hypoadrenocorticism in the standard poodle. J Small Anim Pract.

[23] Oberbauer AM, Bell JS, Belanger JM, Famula TR (2006). Genetic evaluation of Addison’s disease in the Portuguese water dog. BMC Vet Res.

[24] Chase K, Sargan D, Miller K, Ostrander EA, Lark KG (2006). Understanding the genetics of autoimmune disease: two loci that regulate late onset Addison’s disease in Portuguese water dogs. Int J Immunogenet.

[25] Hughes AM, Nelson RW, Famula TR, Bannasch DL (2007). Clinical features and heritability of hypoadrenocorticism in Nova Scotia duck tolling retrievers: 25 cases (1994–2006). J Am Vet Med Assoc.

[26] Boag AM, Catchpole B (2014). A review of the genetics of hypoadrenocorticism. Top Companion Anim Med.

[27] Massey J, Boag AM, Short AD, Scholey RA, Henthorn PS, Littman MP (2013). MHC class II association study in eight breeds of dog with hypoadrenocorticism. Immunogenetics.

[28] Hughes AM, Jokinen P, Bannasch DL, Lohi H, Oberbauer AM (2010). Association of a dog leukocyte antigen class II haplotype with hypoadrenocorticism in Nova Scotia duck tolling retrievers. Tissue Antigens.

[29] Gershony LC, Belanger JM, Short AD, Le M, Hytönen MK, Lohi H (2019). DLA class II risk haplotypes for autoimmune diseases in the bearded collie offer insight to autoimmunity signatures across dog breeds. Canine Genet Epidemiol.

[30] Safra N, Pedersen NC, Wolf Z, Johnson EG, Liu HW, Hughes AM (2011). Expanded dog leukocyte antigen (DLA) single nucleotide polymorphism (SNP) genotyping reveals spurious class II associations. Vet J.

[31] Seddon JM, Berggren KT, Fleeman LM (2010). Evolutionary history of DLA class II haplotypes in canine diabetes mellitus through single nucleotide polymorphism genotyping. Tissue Antigens.

[32] Matzaraki V, Kumar V, Wijmenga C, Zhernakova A. The MHC locus and genetic susceptibility to autoimmune and infectious diseases. Genome Biol. 2017;18. 10.1186/s13059-017-1207-1. 10.1186/s13059-017-1207-1 PMC5406920 28449694

[33] Hughes AM, Bannasch DL, Kellett K, Oberbauer AM (2011). Examination of candidate genes for hypoadrenocorticism in Nova Scotia duck tolling retrievers. Vet J.

[34] Short AD, Boag A, Catchpole B, Kennedy LJ, Massey J, Rothwell S (2013). A candidate gene analysis of canine Hypoadrenocorticism in 3 dog breeds. J Hered.

[35] Short AD, Catchpole B, Boag AM, Kennedy LJ, Massey J, Rothwell S (2014). Putative candidate genes for canine hypoadrenocorticism (Addison’s disease) in multiple dog breeds. Vet Rec.

[36] Short AD, Saleh NM, Catchpole B, Kennedy LJ, Barnes A, Jones CA (2010). CTLA4 promoter polymorphisms are associated with canine diabetes mellitus. Tissue Antigens.

[37] Threlfall AJ, Boag AM, Soutter F, Glanemann B, Syme HM, Catchpole B (2015). Analysis of DLA-DQB1 and polymorphisms in CTLA4 in cocker spaniels affected with immune-mediated haemolytic anaemia. Canine Genet Epidemiol.

[38] Wigginton JE, Cutler DJ, Abecasis GR (2005). A note on exact tests of hardy-Weinberg equilibrium. Am J Hum Genet.

[39] Anderson CA, Pettersson FH, Clarke GM, Cardon LR, Morris AP, Zondervan KT (2010). Data quality control in genetic case-control association studies. Nat Protoc.

[40] Wittke-Thompson JK, Pluzhnikov A, Cox NJ (2005). Rational inferences about departures from hardy-Weinberg equilibrium. Am J Hum Genet.

[41] Yu K, Li Q, Bergen AW, Pfeiffer RM, Rosenberg PS, Caporaso N (2009). Pathway analysis by adaptive combination of P-values. Genet Epidemiol.

[42] Juran BD, Atkinson EJ, Larson JJ, Schlicht EM, Liu X, Heathcote EJ (2010). Carriage of a tumor necrosis factor polymorphism amplifies the cytotoxic T-lymphocyte antigen 4 attributed risk of primary biliary cirrhosis: evidence for a gene-gene interaction. Hepatology.

[43] Barreto M, Santos E, Ferreira R, Fesel C, Fontes MF, Pereira C (2004). Evidence for CTLA4 as a susceptibility gene for systemic lupus erythematosus. Eur J Hum Genet.

[44] Schmidt D, Wilson MD, Ballester B, Schwalie PC, Brown GD, Marshall A (2010). Five-vertebrate ChlP-seq reveals the evolutionary dynamics of transcription factor binding. Science.

[45] Dermitzakis ET, Clark AG (2002). Evolution of transcription factor binding sites in mammalian gene regulatory regions: conservation and turnover. Mol Biol Evol.

[46] Ziegler SF (2006). FOXP3 of mice and men. Annu Rev Immunol.

[47] Bickhart DM, Liu GE (2013). Identification of candidate transcription factor binding sites in the cattle genome. Genom Proteomic Bioinforma.

[48] Whiteld TW, Wang J, Collins PJ, Partridge EC, Aldred SF, Trinklein ND (2012). Functional analysis of transcription factor binding sites in human promoters. Genome Biol.

[49] Bartůněk P, Králová J, Blendinger G, Dvořák M, Zenke M. GATA-1 and c-myb crosstalk during red blood cell differentiation through GATA-1 binding sites in the c-myb promoter. 2003;22:1927–35. 10.1038/sj.onc.1206281. 10.1038/sj.onc.1206281 12673198

[50] Fu W, Ergun A, Lu T, Hill JA, Haxhinasto S, Fassett MS (2012). A multiply redundant genetic switch “locks in” the transcriptional signature of regulatory T cells. Nat Immunol.

[51] Haaning Andersen AD, Lange M, Lillevang ST (2003). Allelic variation of the inducible costimulator (ICOS) gene: detection of polymorphisms, analysis of the promoter region, and extended haplotype estimation. Tissue Antigens.

[52] Neefjes J, Jongsma MLM, Paul P, Bakke O, Jongsma MLM. Towards a systems understanding of MHC class I and MHC class II antigen presentation. Nat Rev Immunol. 2011. 10.1038/nri3084. 10.1038/nri3084 22076556

[53] Macleod D, Charlton J, Mullins J, Bird AP (1994). Sp1 sites in the mouse aprt gene promoter are required to prevent methylation of the CpG island. Genes Dev.

[54] Wolberger C (1998). Combinatorial transcription factors. Curr Opin Genet Dev.

[55] Sadlon TJ, Wilkinson BG, Pederson S, Brown CY, Bresatz S, Gargett T (2010). Genome-wide identification of human FOXP3 target genes in natural regulatory T cells. J Immunol.

[56] Wu H, Peisley A, Graef IA, Crabtree GR (2007). NFAT signaling and the invention of vertebrates. Trends Cell Biol.

[57] Gibson HM, Hedgcock CJ, Aufiero BM, Wilson AJ, Hafner MS, Tsokos GC (2007). Induction of the CTLA-4 gene in human lymphocytes is dependent on NFAT binding the proximal promoter. J Immunol.

[58] Wu Y, Borde M, Heissmeyer V, Feuerer M, Lapan AD, Stroud JC (2006). FOXP3 controls regulatory T cell function through cooperation with NFAT. Cell.

[59] Hoppler S, Kavanagh CL (2007). Wnt signalling: variety at the core. J Cell Sci.

[60] Ioannidis V, Beermann F, Clevers H, Held W (2001). The beta-catenin-TCF-1 pathway ensures CD4 (+) CD8 (+) thymocyte survival. Nat Immunol.

[61] Xue H-H, Zhao D-M (2012). Regulation of mature T cell responses by the Wnt signaling pathway. Ann N Y Acad Sci.

[62] Shah KV, Chien AJ, Yee C, Moon RT (2008). CTLA-4 is a direct target of Wnt/β-catenin signaling and is expressed in human melanoma tumors. J Invest Dermatol.

[63] Chistiakov DA, Savost’anov KV, Turakulov RI, Efremov IA, Demurov LM (2006). Genetic analysis and functional evaluation of the C/T (−318) and a/G(−1661) polymorphisms of the CTLA-4 gene in patients affected with graves’ disease. Clin Immunol.

[64] Wang XB, Zhao X, Giscombe R, Lefvert AK (2002). A CTLA-4 gene polymorphism at position−318 in the promoter region affects the expression of protein. Genes Immun.

[65] Kavvoura FK, Akamizu T, Awata T, Ban Y, Chistiakov DA, Frydecka I (2007). Cytotoxic T-lymphocyte associated antigen 4 gene polymorphisms and autoimmune thyroid disease: a meta-analysis. J Clin Endocrinol Metab.

[66] Chang M-C, Chang Y-T, Tien Y-W, Liang P-C, Jan I-S, Wei S-C (2007). T-cell regulatory gene CTLA-4 polymorphism/haplotype association with autoimmune pancreatitis. Clin Chem.

[67] Kavvoura FK, Ioannidis JPA (2005). CTLA-4 gene polymorphisms and susceptibility to type 1 diabetes mellitus: a HuGE review and meta-analysis. Am J Epidemiol.

[68] Tang S, Tang H, Zhang Q, Wang C, Wang Y, Peng W (2012). Association of cytotoxic T-lymphocyte associated antigen 4 gene polymorphism with type 1 diabetes mellitus: a meta-analysis. Gene.

[69] Wicker LS, Chamberlain G, Hunter K, Rainbow D, Howlett S, Tiffen P (2004). Fine mapping, gene content, comparative sequencing, and expression analyses support Ctla4 and Nramp1 as candidates for Idd5.1 and Idd5.2 in the nonobese diabetic mouse. J Immunol.

[70] Alexander RP, Fang G, Rozowsky J, Snyder M, Gerstein MB (2010). Annotating non-coding regions of the genome. Nat Rev Genet.

[71] Ueda H, Howson JM, Esposito L, Heward J, Snook H, Chamberlain G (2003). Association of the T-cell regulatory gene CTLA4 with susceptibility to autoimmune disease. Nature.

[72] Sutter NB, Ostrander EA (2004). Dog star rising: the canine genetic system. Nat Rev Genet.

